# Metagenomic Analysis of the Airborne Environment in Urban Spaces

**DOI:** 10.1007/s00248-014-0517-z

**Published:** 2014-10-29

**Authors:** Nicholas A. Be, James B. Thissen, Viacheslav Y. Fofanov, Jonathan E. Allen, Mark Rojas, George Golovko, Yuriy Fofanov, Heather Koshinsky, Crystal J. Jaing

**Affiliations:** 1 Physical and Life Sciences Directorate, Lawrence Livermore National Laboratory, Livermore, CA 94551 USA; 2 Eureka Genomics, Hercules, CA USA; 3 Computation/Global Security Directorates, Lawrence Livermore National Laboratory, Livermore, CA USA; 4 Sealy Center for Structural Biology and Molecular Biophysics, University of Texas Medical Branch, Galveston, TX USA

**Keywords:** Aerosol microbiology, Urban air, Airborne bacteria, Metagenomics, Microbiome

## Abstract

The organisms in aerosol microenvironments, especially densely populated urban areas, are relevant to maintenance of public health and detection of potential epidemic or biothreat agents. To examine aerosolized microorganisms in this environment, we performed sequencing on the material from an urban aerosol surveillance program. Whole metagenome sequencing was applied to DNA extracted from air filters obtained during periods from each of the four seasons. The composition of bacteria, plants, fungi, invertebrates, and viruses demonstrated distinct temporal shifts. *Bacillus thuringiensis* serovar *kurstaki* was detected in samples known to be exposed to aerosolized spores, illustrating the potential utility of this approach for identification of intentionally introduced microbial agents. Together, these data demonstrate the temporally dependent metagenomic complexity of urban aerosols and the potential of genomic analytical techniques for biosurveillance and monitoring of threats to public health.

**Electronic supplementary material:**

The online version of this article (doi:10.1007/s00248-014-0517-z) contains supplementary material, which is available to authorized users.

## Introduction

Airborne microbial communities have not been as well characterized as organisms present in soil or aquatic environments [1–4], in part due to the difficulty associated with obtaining sufficient material for study. Previous studies have performed the culture-based examination of airborne microbial populations relevant to agricultural environments [5, 6], city settings [7, 8], and meteorological conditions [9, 10]. Many aerosolized organisms may, however, be difficult to culture, and there has been an increasing interest and an expanding technical capacity for metagenomic surveys of aerosol environments. A number of such studies have been applied to aerosol microbes with potential human contact, such as those in indoor spaces [11–13], suburban and city regions [14, 15], and industrial food and waste facilities [16–18]. These studies have revealed that microbial composition varies depending on many factors, including time, location, and human and animal behavior.

Aerosolized microorganisms are also capable of transcontinental spread [19, 20]. While not all transported organisms may affect human health, recent studies suggest the possibility that transoceanic aerosols could bear etiologic agents of human disease [21]. Aerosolized microorganisms would be difficult or impossible to control, particularly in densely populated urban areas; thus, analytical protocols capable of surveying these populations are of interest for public health and national defense.

Biosurveillance technologies are an important part of the national strategy to prevent the dissemination of a pathogenic biological agent. Toward this end, the U.S. Department of Homeland Security has been operating the BioWatch program, a network of systems for environmental monitoring [22]. BioWatch collects samples from ambient air and is designed to function as an early warning apparatus for local officials and public health authorities. We sought to leverage this collection framework to perform a seasonal survey of the microbial aerosol communities associated with the national capital area. An additional interest of this study was to determine the efficacy of next-generation sequence analysis for identifying a particular agent within an aerosol background. We therefore examined collection filters known to be exposed to *Bacillus thuringiensis* serovar *kurstaki* spores, which are commonly applied as an insecticide against gypsy moths [23], to determine whether *B. thuringiensis* could be detected during dispersal periods.

We used whole metagenome next-generation sequencing to evaluate the sequence content captured by aerosol collectors as comprehensively as possible. While the majority of previous environmental studies have applied 16S ribosomal DNA-based sequencing approaches, we used whole metagenome sequencing to increase the degree of taxonomic resolution and facilitate the identification of non-chromosomal sequences, which could improve species-level characterization and identify viruses, fungi, and plants.

## Methods

### Extraction and Purification from BioWatch Filters

Archived portable sampling unit (PSU) filters were obtained from the Washington D.C. National Capital Region. Filters were collected every day during a 1-week period from each season: winter (January 22–28, 2009), spring (April 20–26, 2009), summer (July 19–25, 2009), and fall (October 25–31, 2009). Filters were also obtained on a day during which *B. thuringiensis* serovar *kurstaki* spores were actively dispersed as a pesticide (May 3, 2007). Eleven filters (corresponding to 11 sampling unit locations) were extracted from every day of the 1-week sampling period, with the exception of the winter, during which only seven of the above sampling unit locations were available for this study. Filters from all locations throughout the 1-week period were combined for extraction. The same locations were surveyed throughout the study period. Filters for some additional locations beyond the 11 noted above were available; however, filters obtained from these locations were obscured by soot, likely due to proximity of the sampling unit to particulate-generating activities. In these cases, the filters could not be used due to the inhibitory effects of particulate matter on DNA amplification. The relative quantity of such soiled filters did not vary according to season. Up to 24 filters were combined per 50-mL conical tube. Thirty milliliters of 100 mM phosphate buffer (pH 7.4) with 0.05 % Tween 80 was added to each tube. Samples were vortexed for 30 s and placed on a rocking shaker for 15 min. The vortexing and rocking process was repeated, in the same buffer, for three additional times. Filters were removed, and the washing buffer was centrifuged to collect the filter material.

DNA purification was performed on the collected pellet using the UltraClean Soil DNA Isolation Kit (MoBio) with some modifications. The pellet extracted from combined filters was resuspended in 100 μL TE buffer, 350 μL MoBio Bead Solution, 60 μL MoBio Solution S1, and 200 μL MoBio Inhibitor Removal Solution. The resuspended pellet was bead beat for 2 min with 0.5-mm zirconia/silica beads and then centrifuged. To the removed supernatant, 250 μL of MoBio Solution S2 was added, incubated at 4 °C for 5 min, and centrifuged at 10,000×*g* for 1 min. Two volumes of MoBio Solution S3 were added to the supernatant. The solution was added to a MoBio spin filter and centrifuged for 1 min at 10,000×*g*, followed by three washes with 300 μL MoBio Solution S4. DNA samples were eluted with 50 μL MoBio Solution S5.

### DNA Amplification

DNA extracted from the samples was amplified using the REPLI-g Midi Kit (Qiagen). This kit performs whole genome amplification using multiple displacement amplification [24, 25]. Samples were amplified for 16 h at 30 °C. Amplified samples were purified using QIAquick PCR Purification columns (Qiagen).

### Sequencing and Quality Control

Two independent sets of the amplified seasonal samples (four samples in each set) were prepared into libraries and sequenced. Each amplified DNA sample in the first set was sequenced on the Illumina Genome Analyzer IIx (GAIIx), using 1 μg DNA for preparation of standard paired-end libraries. Briefly, DNA was fragmented, end repaired, A′ tagged, ligated to adaptors, size selected, and enriched with 18 PCR cycles (the spring sample was enriched with 13 cycles of PCR). Each amplified DNA sample in the second set was sequenced on the Illumina HiSeq 2000, using 300 ng DNA for preparation of paired-end, indexed libraries, as described above.

The spring sample from the first set of the seasonal samples was sequenced on the GAIIx using 51-bp paired-end reads, while the summer, fall, and winter samples from the first set were sequenced as 112-bp paired-end reads on the GAIIx (one sample per lane). All the seasonal samples from the second set were sequenced using 50-bp single-end reads on the HiSeq 2000. Equivalent amounts of the four libraries were multiplexed in one flow cell lane.

Samples exposed to *B. thuringiensis* serovar *kurstaki* were sequenced using 51-bp paired-end reads on the GAIIx. The resultant sequence reads were processed using the default parameters of the Illumina CASAVA pipeline and checked for quality issues. All reads were determined to be of sufficient quality to proceed with subsequent analysis.

### Sequence Read Analysis

Genomic composition of each sample was determined by mapping its sequence reads against viral, bacterial, and eukaryotic (excluding human) sequences (NCBI GenBank database as of March 1, 2013) using Bowtie (version 0.12.7) with up to three mismatches. Because a top-hit-only approach carries a risk of producing false-positive hits (read mapping to a relative of the organism present in the sample, rather than the actual organism), all hits (up to three mismatches) produced by the alignment program were kept and analyzed.

The resulting output from each Bowtie run was parsed to obtain the taxonomy IDs and the names of the organisms that were matched by each read. All possible hits for each read were recorded and classified on the basis of their taxonomic classification using the NCBI taxonomic ID. To avoid bias resulting from overrepresentation of certain species within GenBank and bias associated with reads present in multiple copies within a genome (e.g., rDNA reads), a read was counted as matching to a given taxonomic ID only once, preventing the artificial inflation of number of mapped reads from species with multiple available sub-strain genomes.

To improve specificity, an additional analysis was performed to identify reads whose alignments were unique to a single species—referred to as “informative reads.” This was particularly helpful in distinguishing between reads mapping to closely related species sharing significant sequence similarity. Additionally, when multiple sub-strains of a given bacterial species were present, the bacterial or viral sub-strains were collapsed, such that reads mapping to sub-strains were instead counted as mapping to parent species. This prevented the erroneous dilution of unique reads for species with many sub-strains.

### Quantitative PCR for *B. thuringiensis* Serovar *kurstaki*

The genome copy number for *B. thuringiensis* serovar *kurstaki* present on aerosol filters was quantified using Taqman (Life Technologies) quantitative PCR (F: AGCGTATGCTCGTCTCAAGTAAAA, R: CCTGCCTTGTGGATCTCTAGC, probe: TGCATCGAACTCAATAAAATATTTGTTTTGGAGGG). A standard curve was constructed ranging from 1 to 100,000 genome copies using *B. thuringiensis* serovar *kurstaki* HD1 genomic DNA and was applied for absolute quantification of the experimental samples. All assays were performed in triplicate.

### Statistical Analysis

The Phyloseq (version 1.6.0) package in R (version 3.0.2) was used for data manipulation and construction of graphics [26]. Principal coordinate analysis-based ordering was used to construct heat maps and ordination plots with the Phyloseq implementation of NeatMap [27]. For each ordination plot, percentage variation explained by each component is given along the axes.

## Results

### Alignment of Urban Airborne Sequence Data

Sequence data obtained from the extracted, amplified aerosol samples, including total reads, unique reads, and informative mapped reads, are given for each seasonal sample in Table 1. Informative reads were defined as unique reads mapping to only one unique taxonomic ID. Informative reads were applied for rank-ordering taxonomic IDs. When assigning relative abundance values to an organism in subsequent analyses, total reads mapped to that taxonomic ID were employed in order to capture the total sequence data derived from that species.

**Table 1:** The number of total and unique reads sequenced and the number of informative reads aligned to taxonomic IDs. Informative reads represent unique reads mapping to only one taxonomic ID

Sample	Total reads sequenced	Unique reads sequenced	% unique	Informative reads mapped
Winter 1	90,454,366	21,722,517	24.01	3,727,579
Winter 2	64,234,662	18,759,050	29.2	2,123,026
Spring 1	85,253,374	66,947,821	78.53	19,124,779
Spring 2	68,588,917	57,647,167	84.05	15,703,546
Summer 1	97,944,883	31,861,677	32.53	4,096,208
Summer 2	51,686,698	23,524,936	45.51	2,657,978
Fall 1	97,854,175	55,640,354	56.86	4,053,402
Fall 2	68,228,814	45,999,051	67.42	2,246,574

Informative read values were comparable between the winter, summer, and fall samples but were observed to be much higher in the samples collected during the spring. This observation was primarily due to elevated levels of plant/fungal sequence material and the larger genome sizes associated with these organisms. Specifically, the increased quantity of informative reads was almost entirely due to mapping of sequence data to *Betula nana* (dwarf birch shrub), to which more than ten million informative reads were assigned in each spring sample.

### Seasonal Distribution of Urban Airborne Sequence Data

**Fig. 1:**
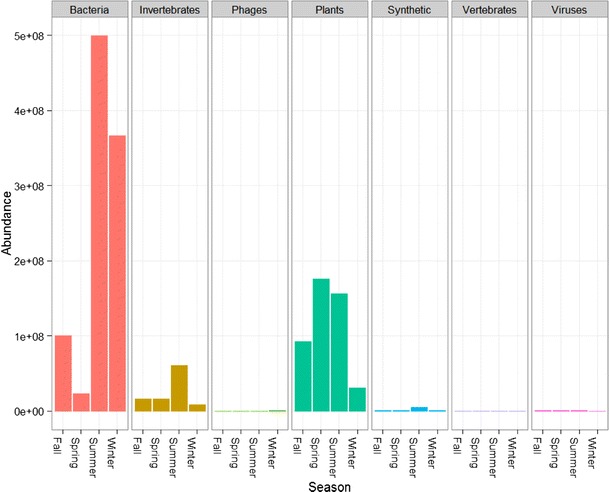
Total sequence data obtained from aerosol material from the four seasons. Material from urban aerosol collection sites was collected, and nucleic acid was extracted and amplified. Whole metagenome sequencing was performed in duplicate, and reads were mapped to bacterial, viral, plant, fungal, and eukaryotic (excluding human) reference genomes. Total reads mapped across both replicates are shown combined in the *plot*, which is segmented according to organism category. Quantity of sequence data (absolute abundance) is shown for each individual season within each category

The distribution of total sequence data (total mapped reads) was examined according to the category of origin (Fig. 1). As was expected, bacterial sequences were featured prominently in these data and observed at the highest levels during the summer, followed by the winter. Sequences associated with plant/fungal material were also frequently observed, peaking during the spring. Sequence data mapping to invertebrate genomes were highest during the summer, remaining relatively consistent in the remainder of the year. Reads were also mapped to phage, sequences annotated as synthetic constructs, vertebrates, and other viruses; however, the sequence content and the number of mapped informative reads were minimal for each of these categories throughout all seasons.

A union set of the top 15 detected genomes, within each season, was compiled and rank ordered based on the number of mapped informative reads (Table 2). This smaller union set was selected to more closely examine the highest abundant organisms present throughout the course of the year. The majority of the top-ranked genomes corresponded to bacteria, largely attributable to soil microbiota. Species commonly associated with skin microflora, including *Staphylococcus* and *Klebsiella*, were also prominently observed. *B. nana* was a dominant contributor to the observed plant sequence data, though this was primarily observed during the spring and fall. The peak in sequence data mapping to invertebrates during the summer was attributed largely to *Aedes aegypti*. Due to the whole metagenome approach, a unique feature of this study was the ability to survey viral content collected by aerosol filters. Although *Pseudomonas* phage was the only virus identified in the top-ranked organisms, a total of 60 viruses were assigned sequence data in this study (Electronic Supplementary Material (ESM) Fig. S1). It is important to note, however, that very few informative reads were assigned to these taxa, indicating that a portion of these alignments may be attributable to other organisms.

**Table 2:** A union set of the top 15 genomes to which sequence reads were aligned. Overall ranking of species is shown according to the number of informative reads mapped. Informative reads are defined as unique reads mapping to only one taxonomic ID

Species	Ranking relative to all detected organisms (ranked by informative read count)							
	Spring		Fall		Winter		Summer	
	1	2	1	2	1	2	1	2
Bacteria								
*Bacillus cereus*	94	24	25	25	19	17	10	8
*Bacillus clausii*	220	224	64	67	150	78	12	15
*Bacillus coagulans*	115	109	53	51	55	51	4	3
*Bacillus megaterium*	30	29	9	8	3	2	2	1
*Bacillus psychrosaccharolyticus*	138	142	98	96	6	5	26	27
*Bacillus pumilus*	165	157	28	29	40	36	9	11
*Bacillus smithii*	143	149	30	32	20	18	5	5
*Bacillus* sp. 1NLA3E	262	305	189	156	9	6	35	32
*Bacillus subtilis*	353	365	142	115	238	204	15	16
*Bacillus thuringiensis*	209	49	66	60	72	89	20	14
Beta proteobacterium FWI2	52	61	19	22	13	14	27	49
*Bradyrhizobium elkanii*	50	78	68	110	10	15	104	275
*Cupriavidus basilensis*	6	6	1	1	1	1	1	4
*Cupriavidus necator*	40	59	17	24	11	11	22	40
*Cupriavidus* sp. HMR-1	12	14	7	7	2	3	6	10
*Geobacillus caldoxylosilyticus*	68	68	40	39	15	16	3	2
*Klebsiella pneumoniae*	38	45	15	20	8	8	13	20
*Ralstonia pickettii*	8	11	6	6	4	4	7	13
*Ralstonia solanacearum*	21	31	8	13	7	10	18	34
*Staphylococcus epidermidis*	14	13	120	104	234	514	17	9
*Staphylococcus hyicus*	88	85	407	2749	263	1799	21	12
*Stenotrophomonas maltophilia*	197	260	34	37	14	12	28	26
Invertebrate								
*Aedes aegypti*	166	460	167	431	109	327	11	7
*Hammondia hammondi*	29	44	41	55	5	7	63	103
*Toxoplasma gondii*	220	301	127	114	98	82	14	17
Phage								
*Pseudomonas* phage F10	727	2335	1572	2749	29	13	748	2666
Plant/fungal								
*Alternaria arborescens*	2	2	3	3	43	950	16	19
*Aureobasidium pullulans*	3	3	22	23	230	866	110	166
*Betula nana*	1	1	4	4	30	207	24	30
*Botryotinia fuckeliana*	9	8	5	5	301	790	276	312
*Castanea mollissima*	10	7	942	1173	940	1799	925	1015
*Cedrus deodara*	582	639	10	9	734	1799	1189	2666
*Chaetomium globosum*	26	30	14	16	12	9	57	78
*Cladosporium sphaerospermum*	7	9	147	169	579	866	119	142
*Cochliobolus sativus*	44	48	11	14	835	1799	32	38
*Gibberella zeae*	11	10	162	135	763	1799	577	763
*Ginkgo biloba*	23	15	248	194	1008	1799	1387	2666
*Hordeum vulgare*	31	37	44	41	17	20	33	29
*Oryza sativa*	15	17	86	91	73	81	186	180
*Penicillium chrysogenum*	13	12	50	47	579	866	112	143
*Pinus taeda*	4	4	16	12	445	1060	161	170
*Prunus persica*	17	16	13	10	802	790	532	433
*Pyrenophora tritici-repentis*	42	46	12	11	608	1060	403	451
*Quercus robur*	5	5	39	33	263	1799	258	530
*Rhizopus microsporus*	436	489	21	15	1008	790	52	43
*Sordaria macrospora*	22	22	2	2	128	128	19	18

### Species-Based Clustering of Seasonal Samples

**Fig. 2:**
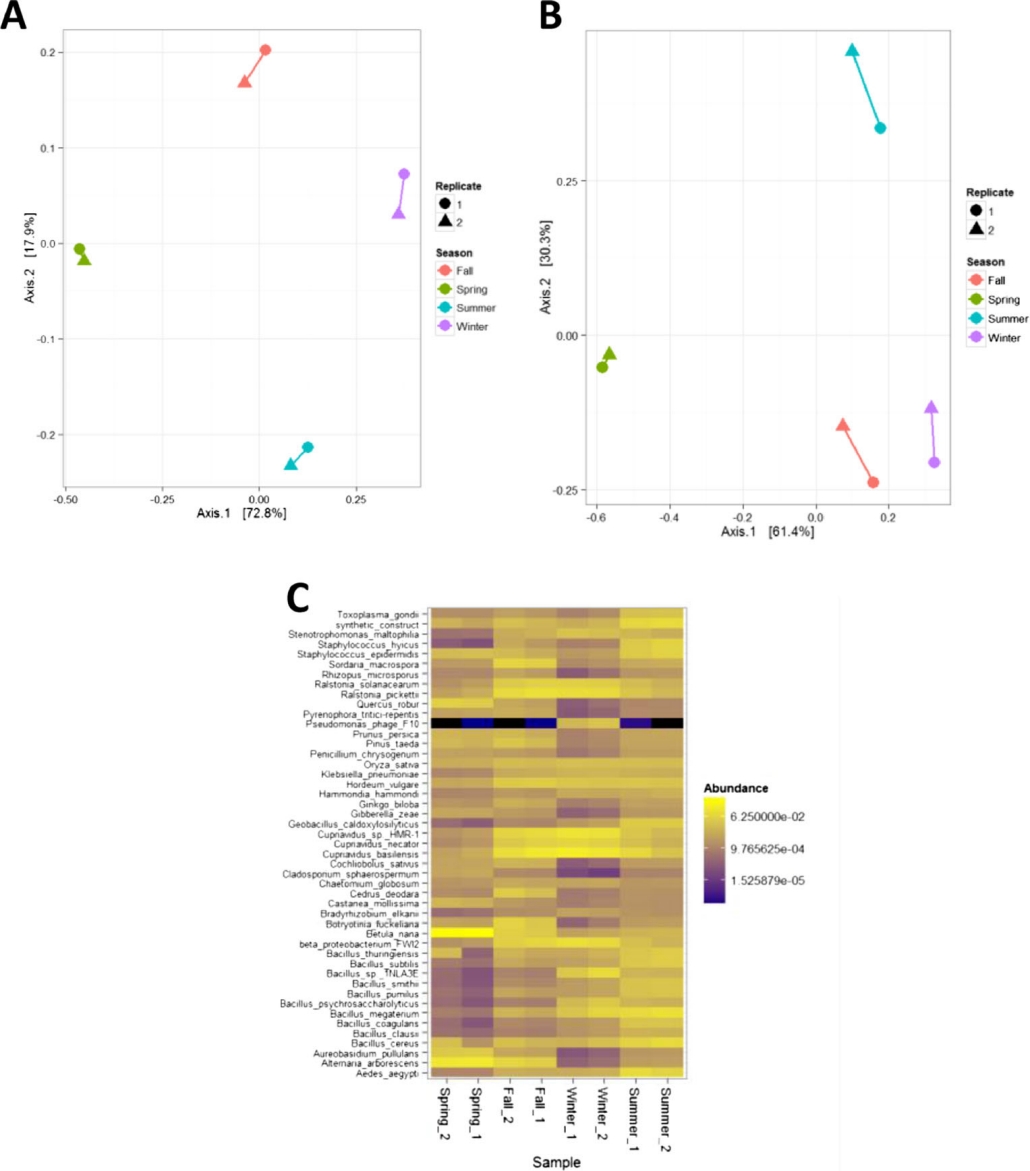
Grouping of airborne seasonal samples according to sequence content. Total sequence data obtained from each seasonal aerosol sample were normalized to obtain relative abundance values. Relative abundances were subjected to principal coordinate analysis, which was applied for grouping individual samples. **a** Ordination plot showing dissimilarity between all seasonal samples, as determined by relative abundance of all taxa. Percent variance is shown *alongside each axis*. **b** A union set composed of the top 15 identified taxa in each sample was constructed based on the number of mapped informative reads (unique read mapping to only one taxonomic ID). The *ordination plot* shows dissimilarity between seasonal samples, as determined using only the union set of high abundant taxa. **c** Heat map displaying only high abundant taxa. Samples are shown *along the horizontal axis* and taxa *along the vertical axis*

Samples from each season were grouped according to the relative abundance of their respective associated species, as measured using the relative number of reads mapped to each taxonomic ID (read counts were normalized to total sequence data for each sample). Clustering was performed using principal coordinate analysis-based ordering in Phyloseq. This grouping was applied both to the full dataset of all detected reference genomes (Fig. 2a) and to a union set of the top 15 taxonomic IDs detected across all seasons (Fig. 2b). Each member of this smaller union set is shown in Fig. 2c. In both cases, duplicates from each season cluster together. When only the top taxonomic IDs were considered, the observed dissimilarity between fall and winter samples was reduced, suggesting that high abundant organisms were more closely shared between these seasons.

**Fig. 3:**
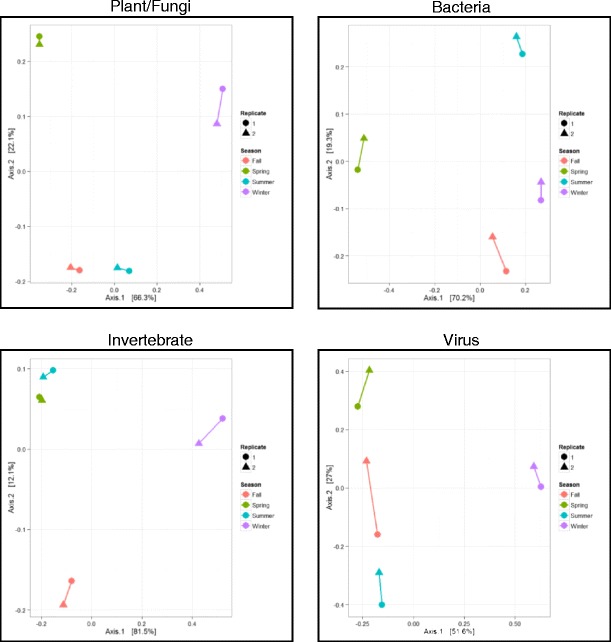
Similarity of airborne seasonal samples based on the sequence content from distinct taxonomic categories. For each seasonal sample, taxa and corresponding relative abundance values were segmented according to the following categories: bacteria, plant/fungal, invertebrate, and virus. Principal coordinate analysis was used to determine dissimilarity between samples, represented in *ordination plots*. Percent variance is given *along each axis*

Relative abundance within the individual bacterial, plant/fungal, invertebrate, and viral categories (corresponding to 3496, 2772, 848, and 60 detected taxa, respectively) was also examined, and sample ordination was performed separately for each category (Fig. 3). In each case, separation was less distinct than was the case when all taxa were examined simultaneously (Fig. 2), although bacterial data facilitated better overall resolution for each season. Specific species categories demonstrated different seasonal separations. For instance, abundance of viral taxa was highly distinct during the winter, while plant/fungal taxa abundance differed greatly during the spring and winter. Notably, the distance between seasons was greater than the distance between individual replicates in each case except virus/phage. These results illustrate that the degree of temporal resolution is dependent on the taxa studied and that combined examination of all available environmental sequence content facilitates higher resolution.

### Detection of *B. thuringiensis* Serovar *kurstaki* in Aerosol Filters

Beyond performing a general survey of seasonal sequence content, an additional interest of this study was the capacity of whole metagenome sequence analysis for identification of a specific organism within an airborne environmental background. Material was obtained from aerosol filters known to be exposed to *B. thuringiensis* serovar *kurstaki* spores, a commonly employed insecticide, and sequencing was performed as previously. Counts for the top 15 detected taxonomic IDs observed in data from aerosol filters are given in ESM Fig. S2. Reads were assigned to multiple *B. thuringiensis* strains, including a large proportion mapping uniquely to *B. thuringiensis* serovar *kurstaki*. Overall, *B. thuringiensis* reads represented 21 % of informative reads in aerosol filters. A large number of reads also mapped to *Bacillus cereus*, a close relative of *B. thuringiensis*. Quantitative PCR was performed to validate and quantify the presence of *B. thuringiensis* serovar *kurstaki*, yielding a Ct value of 22.6 ± 0.1. This value corresponded to a copy number of 6.01 × 10^4^ genome copies per aerosol filter or 4.91 × 10^3^ copies/cm^2^. These data demonstrate the effective extraction and detection of *B. thuringiensis* serovar *kurstaki* spores.

## Discussion

Airborne microbial communities bear relevance to both environmental and health safety concerns, particularly those populations associated with urban areas. The aerosol samples in this study allowed both for holistic examination of taxa and a case study for detection of a pathogen surrogate (*B. thuringiensis* serovar *kurstaki*) within an urban aerosol background. Current detection methods rely largely on multiplex PCR assay panels, which are designed to detect a specific subset of biological agents. This study demonstrates that analyses of shotgun metagenomic sequence data are capable of identifying specific pathogens of interest and could complement these existing technologies. In the case of a detection event, sequence data could provide supplemental information such as strain identity and antimicrobial resistance factors. Further, the unbiased whole metagenome approach allows for potential detection of unknown pathogens for which existing assays may not be designed.

To characterize the general airborne content across each of the four seasons, the material from aerosol filters collected from the National Capital Region during the winter, spring, summer, and fall was extracted and sequenced. A whole metagenome approach was applied to capture all potential bacterial, viral, invertebrate, and plant sequences as well as to facilitate deeper taxonomic resolution.

Due to low available biomass, multiple displacement amplification (MDA) was performed prior to sequencing. Although whole genome amplification (WGA) does risk the introduction of bias in amplifying the microbial community, the MDA protocol used in this study has been demonstrated to be less biased in microarray-based microbial community analysis [24]. MDA-based protocols have been shown to result in less bias than other whole genome amplification methods, as determined by high-throughput sequencing of microbial genomes [28]. Commonly applied MDA kits have also been shown to result in uniform amplification through array-based analysis of DNA copy number variations [25]. It is, however, important to note that all amplification methods will impart some bias. Recent studies have demonstrated that WGA methods are influenced by DNA quality and size as well as GC content [29] and that observed microbial gene content may be biased by MDA [30]. While WGA, and specifically MDA, enables the unique examination of low biomass samples, experiments such as the studies cited above reinforce the importance of interpreting resultant data carefully, particularly when DNA integrity and GC content are expected to vary widely.

The ranking of bacterial taxa belonging to the genera *Ralstonia*, *Cupriavidus*, and *Bacillus* remained consistently high throughout the year, likely due to the fact that many of these organisms are ubiquitously found in soil. Among such species are *Ralstonia pickettii*, *Ralstonia solanacearum*, and *Cupriavidus necator*, which are not associated with human disease, although there have been reported cases of hospital-acquired *R. pickettii* infection [31]. Consistent observance of these organisms is likely due to continuous aerosolization from local soil reservoirs.

Members of the *Bacillus* genera, also commonly observed in soil, were similarly observed throughout the year. *B. cereus* and *B. thuringiensis*, both detected in this study, are closely related to *Bacillus anthracis*, the causative agent of anthrax [32]. Their presence could be highly relevant when attempting to identify *B. anthracis* specifically [33]. The increased frequency of *B. thuringiensis* during summer months is likely due to its noted insecticidal use. Many of the above noted bacteria maintained their presence during the winter months, despite the expected reduction in the aerosolization of microbe-laden terrestrial material. Previous studies have, however, observed elevated levels of certain bacterial groups during colder months, particularly those belonging to Bacteroides, Firmicutes, and Fusobacteria, which was attributed to aerosolized animal fecal material [15].

*Staphylococcus* ranking peaked during the summer and spring in our data, with comparatively low levels being observed during the winter and fall. *Staphylococcus*, in particular *Staphylococcus epidermidis*, are commonly found in the human skin microflora, with pathogenicity of staphylococcal infections varying widely dependent on strain, site of colonization, and host immune competence. Observation of *Staphylococcus* in urban aerosols could be due to aerosolization of human microflora. Reduced incidence during the winter and fall may be due to a limited epidermal exposure during colder months as well as a higher incidence of staphylococcal infections during the warm season [34].

Other primary contributors to sequence data obtained from urban aerosols included plants and fungi. This was particularly true during the spring, during which *B. nana* and *Pinus taeda* were the top-ranked plant taxa detected. These observations were expected, given increased levels of pollination during these time periods. *Alternaria arborescens* and *Aureobasidium pullulans* were the highest ranked fungal species detected and were likely collected simultaneously with associated plant material, as these organisms exist primarily as a plant pathogen and epiphyte, respectively.

The majority of observed viruses in this study were likely collected concurrently with specific bacterial or human content, such as bacteriophage and human-associated viral nucleic acid (herpesviruses, endogenous retroviruses). The seasonal patterns observed based on viral content may be reflective of these relationships. Given that our methodology was not expressly designed for viral particle collection, it is likely that a significant portion of airborne viruses were not isolated. A previous study specifically designed for collection of airborne viral content in the near-surface atmosphere did observe temporal patterns in viral composition, dependent on both temperature and humidity [35]. As expected, this previously published study yielded more extensive and diverse viral communities.

When taxonomic abundance data were employed for ordination of seasonal samples, distinct grouping of these samples was observed. It was also found that the use of a more taxonomically inclusive profile yielded improved seasonal resolution. Previous examination of the microbial composition in near-surface aerosols in the summer and winter similarly observed a correlation of samples by season [15] as well as according to land use characteristics at the collection location [14]. Further, recent work showed that the prevalence of rare, low-abundant taxa in a variety of environmental (including air) and clinical samples varied dramatically over time, disproportionately contributing to temporal changes in diversity [36]. Such seasonal correlation and annual recurrence of microbial communities have also been observed in remote, high altitude regions [37]. Spatial information was not available for the samples in this study; however, geographically dependent variations in the airborne microbial community structure have been previously observed [14, 38]. Observations from these studies, in combination with the temporal clustering observed in the current study, suggest the possibility that metagenomic profiles could potentially be useful for tracing samples with distinct origins.

In addition to assessing general aerosol content, a further aim of this study was to determine whether intentional aerosol release of an organism could be detected by metagenomic sequence analysis. Toward this end, the material known to be exposed to the aerosolized pesticide, *B. thuringiensis* serovar *kurstaki*, was subjected to sequencing. Sequence analysis did detect the presence of *B. thuringiensis*, confirming the capacity of our metagenomic sequence analysis approach for detecting a spore-borne aerosolized biological agent. In the actual event of detection of such an agent, further assays such as quantitative PCR could be performed for subsequent validation.

A sequencing-based approach may not be practical in all instances due to the extended time required for characterization. Further, although low biomass samples may be effectively examined via WGA with nanogram starting quantities of DNA, these techniques are costly to perform. It is, therefore, likely that sequencing would be applied as a complementary approach or if higher resolution analysis is required for detailed genomic inquiry. Sequencing throughput and costs are, however, scaling rapidly; thus, it is likely that the described protocols could approach standard practice with technical advancement and analytical automation for specific applications.

In summary, our data represent a broad seasonal survey of an urban aerosol bioburden, providing a temporal indication of the airborne metagenomic complexity in this space. Such data could be valuable in discerning whether a detected organism or profile is distinct from the microbial background. The ability to survey the airborne metagenome, as well as the application of targeted detection within this background, could provide a valuable molecular biosurveillance tool for monitoring potential threats to public health.

## Electronic supplementary material

Below is the link to the electronic supplementary material.

Fig. S1 Viral sequence content in airborne seasonal samples. Seasonal samples were grouped according to sequence data mapping to viral taxa only. Groupings are represented in a heatmap showing samples along the horizontal axis and viral taxa along the vertical axis.

Fig. S2 *Bacillus thuringiensis* kurstaki identification by sequencing. Nucleic acid was extracted from aerosol filters known to have been exposed to *B. thuringiensis* kurstaki spores, followed by sequencing and quantification of informative reads. The top 15 microbial taxa to which reads were uniquely mapped were identified. *B. thuringiensis* is highlighted in the plot using red bars.
