# Supplementary figures and images for: Metagenomic Analysis of the Airborne Environment in Urban Spaces
Source: Microb Ecol. 2014 Oct 29;69(2):346–55. doi: 10.1007/s00248-014-0517-z (PMC4312561; doi:10.1007/s00248-014-0517-z)

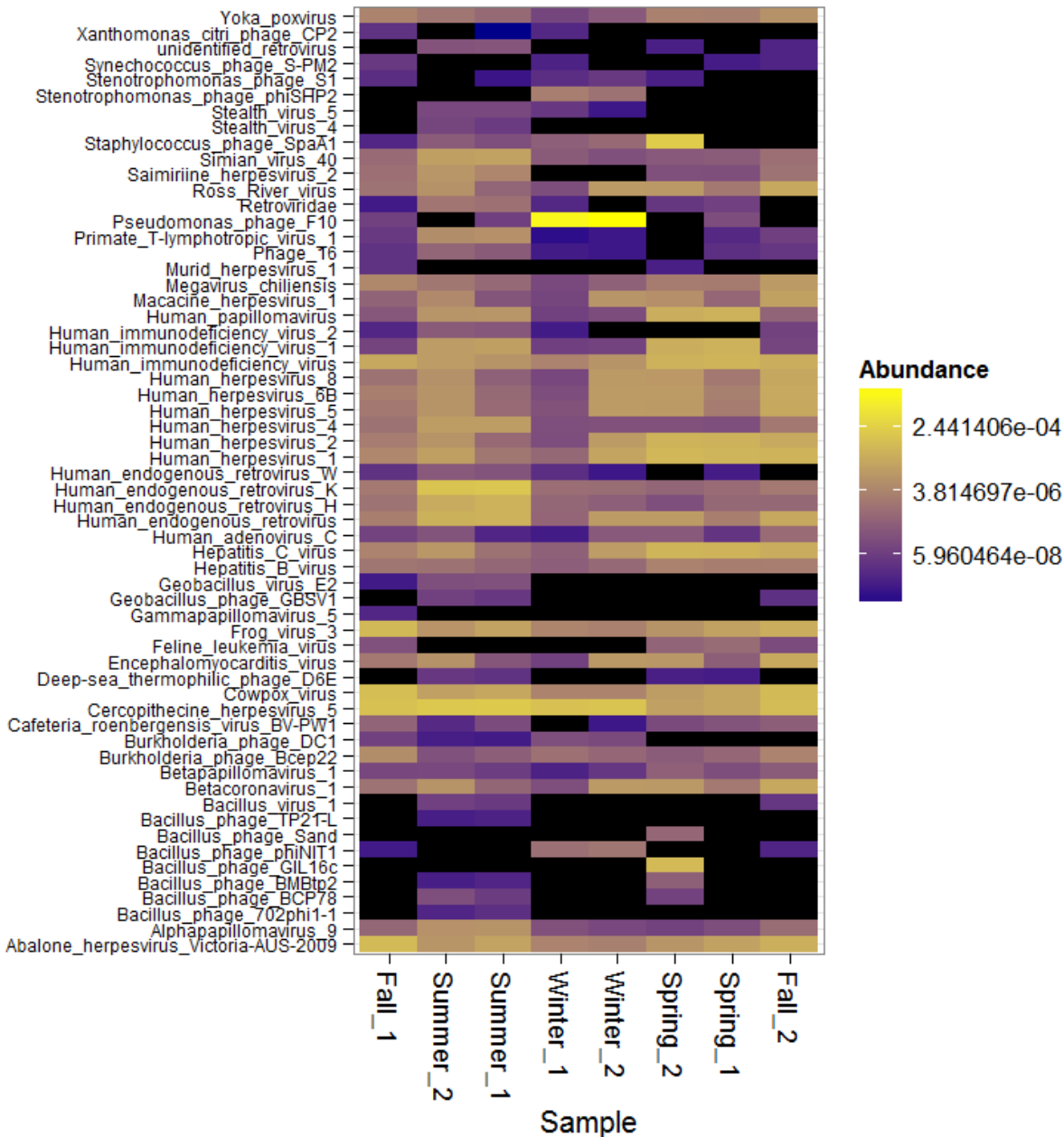

Supplement: Supplementary file 1 — Viral sequence content in airborne seasonal samples. Seasonal samples were grouped according to sequence data mapping to viral taxa only. Groupings are represented in a heatmap showing samples along the horizontal axis and viral taxa along the vertical axis. [file 248_2014_517_MOESM1_ESM.pdf]

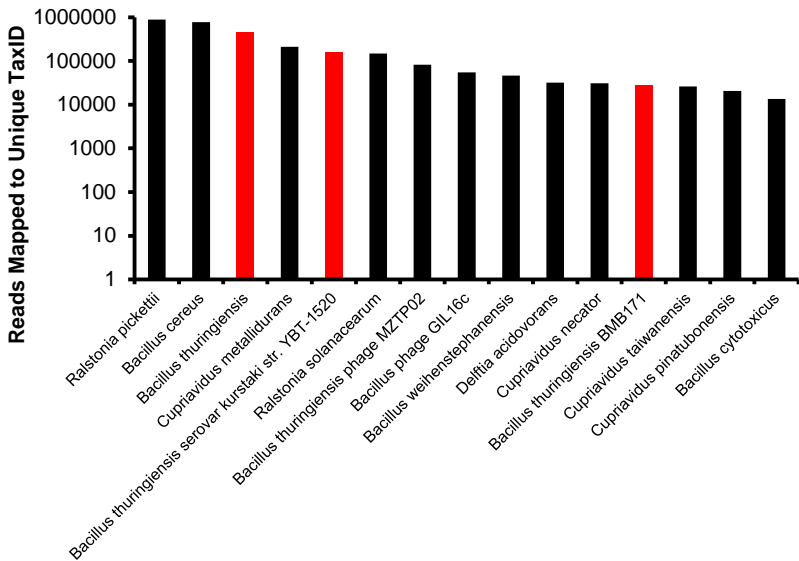

Supplement: Supplementary file 2 — Bacillus thuringiensis kurstaki identification by sequencing. Nucleic acid was extracted from aerosol filters known to have been exposed to B. thuringiensis kurstaki spores, followed by sequencing and quantification of informative reads. The top 15 microbial taxa to which reads were uniquely mapped were identified. B. thuringiensis is highlighted in the plot using red bars. [file 248_2014_517_MOESM2_ESM.pdf]
